# A Case of Recurrent Aspiration Pneumonia and Hyperosmolar Hyperglycemic State Following Severe Stroke and Its Management With Radiologically Inserted Gastrostomy (RIG) Tube Placement

**DOI:** 10.7759/cureus.86846

**Published:** 2025-06-27

**Authors:** Zain Asif

**Affiliations:** 1 Stroke Medicine, Fairfield General Hospital, Bury, GBR

**Keywords:** hyperosmolar hyperglycemic state (hhs), percutaneous endoscopic gastrostomy (peg) feeding, recurrent aspiration pneumonia, s: acute ischaemic stroke, stroke

## Abstract

This report describes the case of a 65-year-old previously healthy man who presented with a severe left pontine ischaemic stroke, resulting in significant neurological deficits and a prolonged hospital stay. He had a pre-morbid modified Rankin Score of 0, with a background of type 2 diabetes mellitus (HbA1c 86 mmol/mol), a history of smoking, recurrent vomiting, and oesophagitis. During admission, he developed frequent episodes of aspiration pneumonia, which significantly complicated his recovery. His Glasgow Coma Scale (GCS) remained low (E4M6V1) throughout, indicating limited neurological improvement. Posterior circulation strokes involving the pons are particularly severe due to the concentration of cranial nerve nuclei and vital autonomic pathways in this region. His clinical course was further complicated by the development of a hyperosmolar hyperglycaemic state (HHS), requiring medical management. As his condition progressed, peripheral venous access became increasingly difficult, eventually necessitating central venous catheter insertion. Following the placement of a radiologically-inserted gastrostomy (RIG) tube, the frequency of aspiration-related chest infections reduced, and his condition temporarily stabilised. However, his overall neurological and functional recovery remained limited. He was eventually discharged to a nursing home but sadly passed away several months later due to ongoing complications. This case highlights the challenges in managing patients with severe brainstem strokes complicated by recurrent aspiration pneumonia, metabolic disturbances such as HHS, and vascular access difficulties. It also reflects the potential role of RIG feeding in reducing respiratory complications in patients with severe dysphagia.

## Introduction

Stroke is a leading cause of long-term disability and mortality, with over 100,000 cases annually in the United Kingdom (UK) and nearly 12 million new strokes globally each year [1,2]. Severe ischaemic strokes, particularly those involving the posterior circulation, are associated with significant neurological deficits and can result in prolonged hospital admissions [3]. Complications such as dysphagia are common and increase the risk of aspiration pneumonia, which can significantly impact recovery [4,5]. Additionally, severe strokes may trigger hyperosmolar hyperglycaemic state (HHS), a metabolic complication requiring careful clinical management [6,7].

Managing patients with severe strokes can be challenging, especially when complicated by recurrent infections, metabolic disturbances, and difficulties with vascular access. Percutaneous endoscopic gastrostomy (PEG) tube placement is often considered in patients with persistent swallowing problems to reduce the risk of aspiration [8]. It is generally the preferred method for long-term enteral feeding due to its safety and ease of placement. However, PEG tubes may be contraindicated in patients with oesophageal ulcers, strictures, or other anatomical difficulties. In such cases, radiologically inserted gastrostomy (RIG) tubes offer a suitable alternative. Inserted under imaging guidance, a RIG tube is often used when endoscopy is not feasible. Studies have shown that RIG can effectively reduce the risk of aspiration and improve nutritional status in patients with severe dysphagia following stroke [9].

This report describes the case of a patient with a severe left pontine infarct whose clinical course was complicated by recurrent chest infections, HHS, and the eventual need for PEG tube insertion during a prolonged hospital stay.

## Case presentation

**Figure 1:**
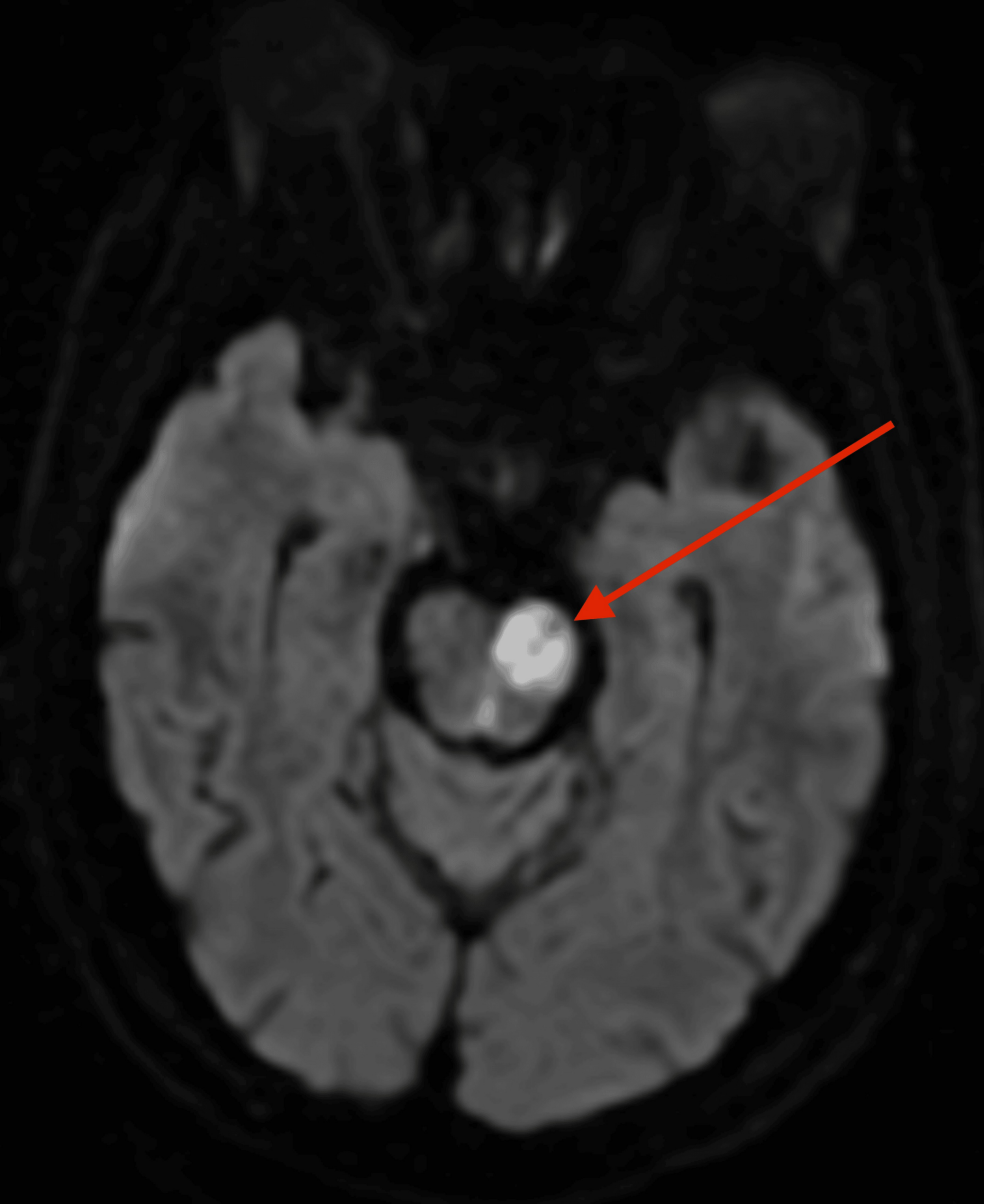
MRI Brain showing left pontine infarct MRI brain showing an area of restricted diffusion in the left anterior pons (indicated by red arrow), consistent with acute ischaemia. Findings are in keeping with a left pontine infarct.

A 65-year-old man with a history of type 2 diabetes mellitus (T2DM) and hypertension was admitted to North Manchester General Hospital on June 30, 2024, with coffee ground vomiting. During his hospital stay, he developed new-onset right-sided upper and lower limb weakness. Magnetic resonance imaging (MRI) of the brain demonstrated an acute infarct involving the left anterior pons, indicated by a red arrow in Figure 1, consistent with a posterior circulation ischaemic stroke. While the MRI findings clearly demonstrated a left pontine infarct consistent with acute ischaemia, other potential differential diagnoses considered included demyelinating lesions, brainstem neoplasms, and infectious or inflammatory processes. However, the acute presentation, clinical course, and imaging characteristics strongly supported ischaemic stroke as the primary diagnosis.

Following initial stabilisation, the patient was repatriated to Fairfield General Hospital on July 5, 2024, for further management and rehabilitation. On clinical examination, he was markedly drowsy with a Glasgow Coma Scale (GCS) of E4M6V1, reflecting eye opening to speech, obeying commands, and absent verbal response likely due to severe dysphasia. His neurological status remained largely unchanged throughout admission, with no significant improvement in GCS observed over time. Functionally, he was profoundly dependent, requiring full nursing care. He experienced recurrent episodes of aspiration pneumonia, requiring multiple courses of intravenous antibiotics and supportive care.

The patient’s clinical course was further complicated by the development of an HHS, despite being on treatment for T2DM prior to admission. His blood sugars were very difficult to control, despite frequent reviews by the Diabetes Specialist Nurse (DSN) team. Biochemical investigations showed a serum osmolality of 356 mOsm/kg and low ketone levels (around 0.4 mmol/L), consistent with HHS. The HHS was likely precipitated by the acute stress of the stroke and recurrent infections. It was managed with fluid resuscitation and a variable rate insulin infusion. As peripheral venous access became increasingly difficult, a peripherally inserted central catheter was inserted via the right basilic vein under ultrasound guidance. We used standard infection prevention measures, including antimicrobial-impregnated dressings, to help reduce the risk of catheter-related infections. This allowed reliable intravenous access for ongoing treatment.

Due to severe oropharyngeal dysphagia seen on videofluoroscopy, PEG placement was initially considered. However, the patient had significant oesophageal problems, including large ulcers and strictures, which made endoscopic passage unsafe and hence contraindicated PEG placement. Therefore, a RIG tube was inserted on August 30, 2024. After RIG placement, the frequency of aspiration-related chest infections noticeably decreased from approximately one episode every two weeks to significantly fewer occurrences.

Despite these interventions, the patient’s neurological recovery remained poor, and he remained fully dependent on nursing care. After a prolonged and medically complex admission, he was discharged home on November 6, 2024, with enteral feeding in place and community support arranged. Sadly, he passed away at home on April 14, 2025, aged 66, due to ongoing complications of his brainstem stroke.

The key events of his clinical course are summarised in Table 1.

**Table 1:** Clinical timeline of key events including the patient’s admission, including diagnosis, complications, interventions, and outcome VRII: variable rate insulin infusion; RIG: radiologically inserted gastrostomy

Date	Event
June 30, 2024	Admitted to North Manchester General Hospital with coffee ground vomiting; developed right-sided weakness
July 4, 2024	MRI brain confirmed an acute infarct in the left anterior pons (posterior circulation stroke)
July 5, 2024	Repatriated to Fairfield General Hospital
July–August 2024	Reduced consciousness (E4M6V1); recurrent aspiration pneumonia episodes
Mid-August 2024	Developed hyperosmolar hyperglycaemic state; managed with VRII and fluids
August 2024	Central venous catheter inserted due to poor peripheral access
August 30, 2024	RIG tube placed following unsafe videofluoroscopy findings
Post-RIG Tube Placement	Reduced frequency of aspiration events
November 6, 2024	Discharged from hospital after prolonged inpatient stay due to left pontine infarct, dysphagia requiring RIG, and multiple medical complications. Referred for community and family support.
April 14, 2025	Passed away at home due to ongoing complications of brainstem stroke

## Discussion

Stroke remains one of the leading causes of long-term disability and mortality worldwide. Posterior circulation strokes, accounting for approximately 20% of all ischaemic strokes, can present with a range of neurological deficits depending on the structures involved [1]. Brainstem infarcts, particularly those affecting the pons, are associated with high morbidity due to the density of critical motor and sensory pathways [2].

Dysphagia is a frequent complication following brainstem strokes, with reported prevalence rates up to 65% [3]. Early identification and management of dysphagia are important to reduce complications in stroke patients. Impaired swallowing increases the risk of aspiration pneumonia, which is a significant contributor to morbidity and mortality in stroke patients [4]. In this case, recurrent aspiration events led to multiple episodes of pneumonia, eventually prompting the placement of a RIG tube. Although enteral feeding reduces the risk of aspiration from oral intake, it does not fully eliminate the risk of aspiration pneumonia due to impaired protective reflexes [5].

The patient’s condition was further complicated by an HHS, which can be triggered by acute illnesses like stroke and infections. Blood tests showed a high serum osmolality of 356 mOsm/kg and consistently low ketone levels (around 0.4 mmol/L), confirming the diagnosis of HHS. Metabolic disturbances such as HHS are known to increase mortality and worsen neurological recovery in stroke patients. Managing this alongside repeated infections, poor oral intake, and problems with venous access made his care especially challenging. Recurrent aspiration pneumonia likely contributed to systemic inflammation and metabolic stress, further impeding neurological recovery and prolonging hospitalisation. Enteral feeding interventions such as PEG and RIG tubes have been shown to reduce aspiration risk and improve nutrition in patients with severe dysphagia [8,9]. Both methods provide similar benefits, with the choice depending on patient-specific factors and contraindications.

Central venous access was required in this case, with appropriate infection prevention measures in place. The combination of severe dysphagia, recurrent aspiration pneumonia, and metabolic instability from HHS created a complex clinical scenario that significantly hindered the patient’s neurological and functional recovery.

## Conclusions

This case highlights the complex clinical course often seen in severe posterior circulation strokes. Early MRI was critical in identifying a left pontine infarct and guiding appropriate care. Brainstem infarcts can cause profound neurological deficits, and dysphagia significantly increases the risk of aspiration pneumonia. While gastrostomy feeding can help reduce this risk, especially with a RIG tube when a PEG tube is not suitable, patients often remain vulnerable to respiratory complications. The development of HHS and the need for central venous access further complicated care. Early recognition of swallowing difficulties, careful metabolic management, and timely planning for long-term intravenous access are key to improving outcomes. This report highlights the role RIG feeding can play when a PEG tube is contraindicated and reinforces the value of early MRI and multidisciplinary input in managing complex stroke patients.
